# Water-soluble and adjustable fluorescence copolymers containing a hydrochromic dye: synthesis, characterization and properties†

**DOI:** 10.1039/c8ra01306c

**Published:** 2018-04-11

**Authors:** Le Ju, Tianyou Qin, Ting Zhang, Peng Wang, Lan Sheng, Sean Xiao-An Zhang

**Affiliations:** College of Chemistry, Jilin University Changchun 130012 China shenglan17@jlu.edu.cn; Department of Chemistry and Pharmacy, Zhuhai College of Jilin University Zhuhai 519041 China; State Key Lab of Supramolecular Structure and Materials, College of Chemistry, Jilin University Changchun 130012 China

## Abstract

Water solubility and adjustable fluorescence properties have been successfully implemented in the hydrochromic amino rhodamine *via* copolymerization. Four copolymers have been synthesized and clearly characterized by UV-Vis spectroscopy, proving greater detail than the commonly used NMR and IR technologies. The four copolymers have good solubility in pure water and in many common organic solvents, while preserving the hydrochromism of the dye monomer. Based on aggregation and dispersion of the copolymers as adjusted by solvent media and temperature, reversible fluorescence properties were successfully realized. Furthermore, their luminescence in solid state was observed. These studies are of great significance for expanding the application of hydrochromic dyes in biological fields and promoting green industrialization.

## Abstract 2

Water solubility and adjustable fluorescence adjustable properties have been successfully endowed to established in a hydrochromic dye *via* copolymerization.

## Graphical abstract

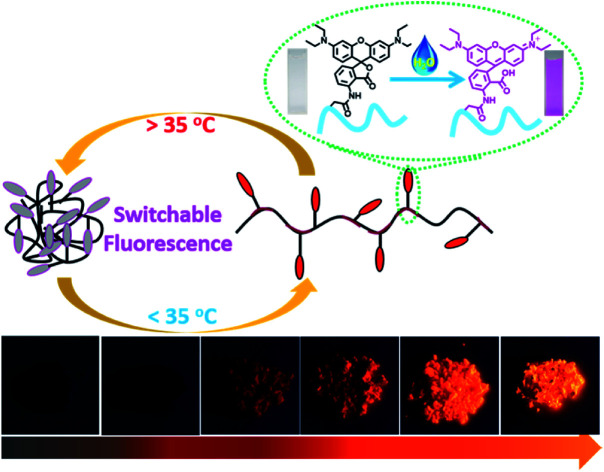

## Introduction

As a source of life, water participates in almost all metabolic processes, supplies solvents to cells and organelles, serves as a lubricant of the organism, and is the foundation for continuance of life.^1^ Inspired from nature and life, scientists are actively exploring excellent water-soluble drugs or materials with the hope of favorable biological applications or green industrial production to better serve human health and life.^2–7^ Recently, hydrochromic materials have received much concern due to their wide application in rewritable imaging on paper,^8,9^ humidity sensors,^10–14^ anti-counterfeit,^15^ encrypting information,^16,17^*etc.* To meet the green industrial demand and to further extend their applications, water-soluble hydrochromic materials with fluorescence are very necessary but rare.

Rhodamine compounds are widely used for dyes^18,19^ and ion detections^20–22^ due to their high molar extinction coefficients, excellent photostability, good fluorescent property with high quantum yields and relative ease of modification. In our previous study, it was found that modifying rhodamine with amino (NH_2_-Rh) can endow it with hydrochromism for application in water-jet rewritable paper.^23^ That is, isomerization of the lactone form of NH_2_-Rh takes place to an open-ring form in the presence of water, showing a bright colour. Conversely, when water disappears, the open-ring structure reversibly returns to its lactone form and the corresponding colour fades simultaneously. However, after modifying with an amino group, the fluorescence of rhodamine was weakened greatly after the addition of water, especially in solid or solid substrate, showing almost no fluorescence.^23^ In order to expand its applications, it is necessary to restore its fluorescence properties, especially in water or in solid state.

Poly(*N*-isopropylacrylamide) (PNIPAM), as a water-soluble and thermosensitive smart polymer, has been applied to drug delivery,^24–26^ controlled-release carriers,^27^ microreactor template synthesis of nanoparticles,^28,29^ and optical materials.^30–32^ It is water soluble due to the strong hydrogen-bonding interaction between *N*-isopropylacrylamide groups and water molecules. When the temperature is higher than its “lower critical solution temperature” (LCST),^33–36^ its solubility is greatly reduced. This thermo-controlled solubility property is reversible and has given PNIPAM a wide range of applications. Fluorescence tags have been copolymerised with PNIPAM^37–39^ and used to elucidate polymer properties;^40,41^ however, the obtained fluorescence response signals are often weak. Thus, copolymers with PNIPAM and high-intensity fluorophores are still being pursued.

In the present work, we have synthesized four copolymers, poly(AM-Rh*_x_ co* NIPAM*_y_*)s, with different ratios of *x* to *y*, by grafting simply modified NH_2_-Rh unit-AM-Rh to water-soluble PNIPAM chains by copolymerization. It is expected that the poly(AM-Rh*_x_ co* NIPAM*_y_*)s will maintain the hydrochromic property of NH_2_-Rh while gaining water solubility and fluorescence.

## Results and discussion

### Synthesis and characterization of poly(AM-Rh*_x_ co* NIPAM*_y_*)

**Scheme 1:**
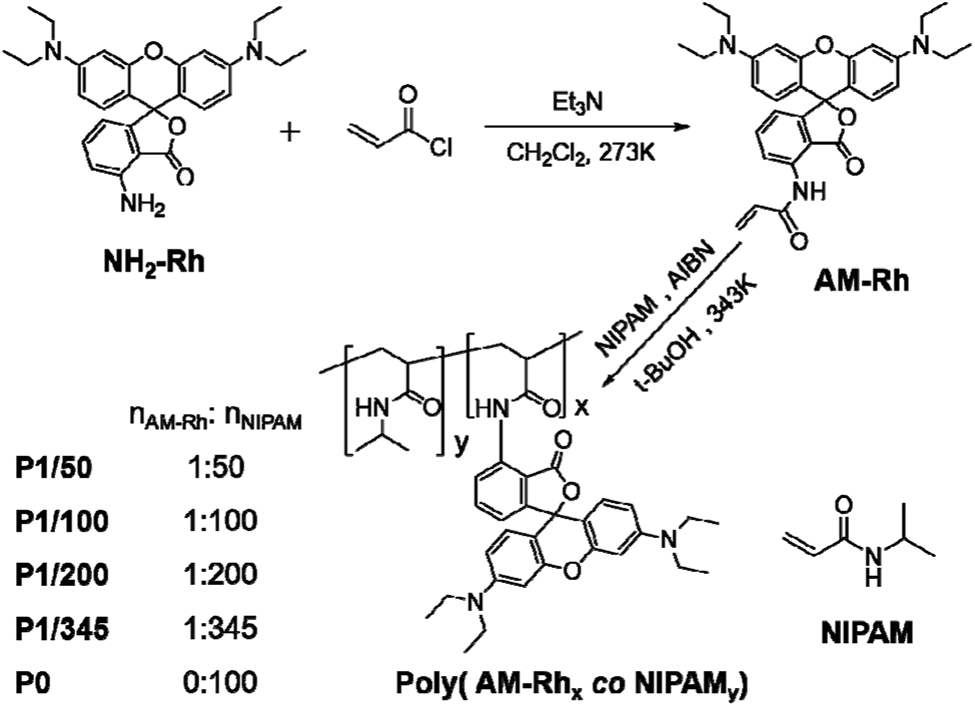
Synthesis of AM-Rh and poly(AM-Rh*_x_ co* NIPAM*_y_*)

The general synthesis route of the poly(AM-Rh*_x_ co* NIPAM*_y_*) copolymers is summarized in Scheme 1. AM-Rh was obtained by acylation reaction of NH_2_-Rh and acryloyl chloride, with 52% yield, and characterized by NMR and MS analysis (see Fig. S1†). Poly(AM-Rh*_x_ co* NIPAM*_y_*)s were successfully synthesized by radical polymerization of NIPAM monomer with AM-Rh as pink solids, which were obtained by precipitation from methanol and diethyl ether. The solids were further purified through dialysis (using a membrane with molecular weight cutoff of 5000) in methanol for 60 h to remove the small residues of AM-Rh and NIPAM. Poly(AM-Rh*_x_ co* NIPAM*_y_*)s with different ratios (*x*/*y*) of the respective units could be regulated by varying the reaction molar ratio of AM-Rh to NIPAM for polymerization. Four copolymers, P1/50, P1/100, P1/200, and P1/345, were prepared with AM-Rh : NIPAM molar ratios of 1 : 50, 1 : 100, 1 : 200, and 1 : 345, respectively. In addition, as a control experiment, poly(NIPAM) (P0) was also prepared.

**Fig. 1:**
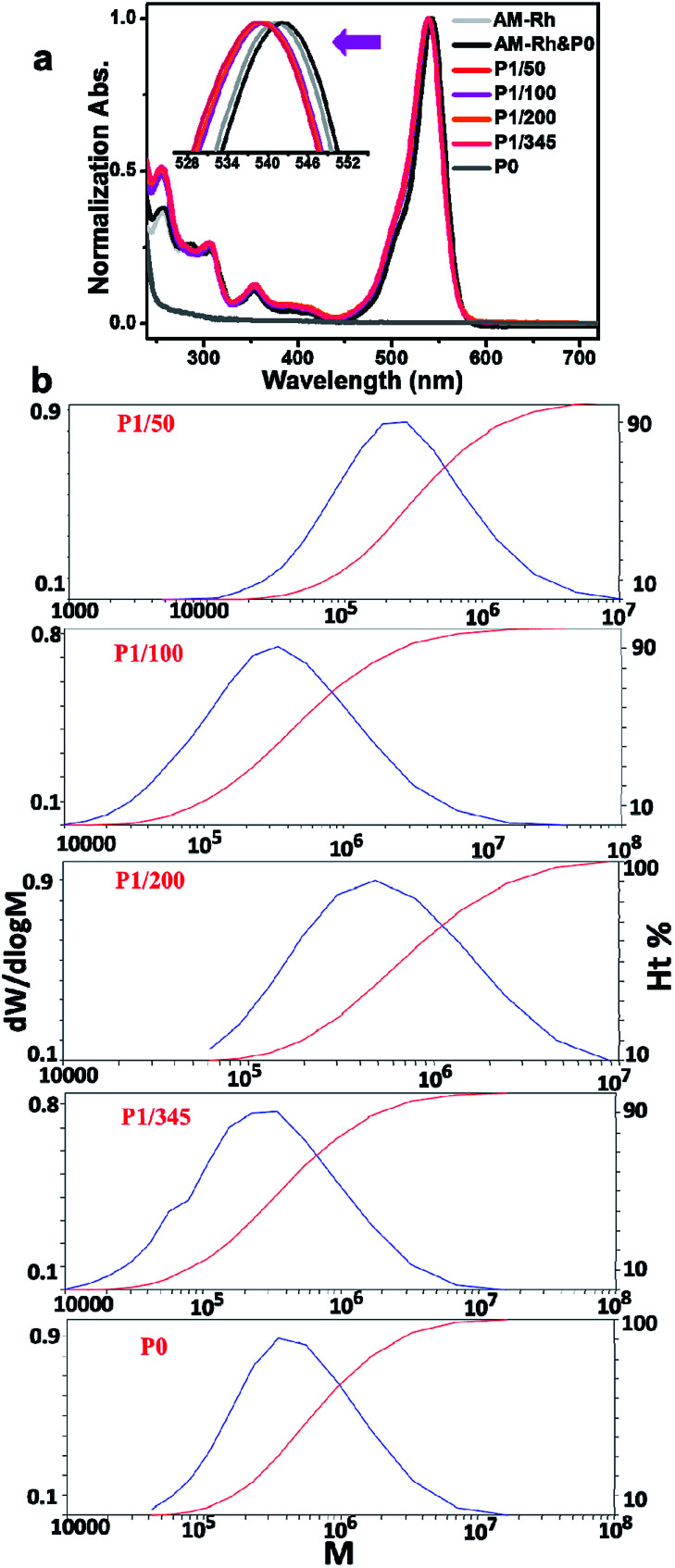
(a) Normalized UV-Vis spectra of P1/50, P1/100, P1/200, P1/345, P0, AM-Rh and mixture of AM-Rh and P0 (AM-Rh & P0) in methanol. (b) The plots of dwdlog M *vs.* M for poly(AM-Rh*_x_ co* NIPAM*_y_*)s and P0.

Characterization of polymers containing functional molecules has always been a difficult problem. NMR and IR technologies are currently used commonly; however, the information indicating bonding between polymers and functional molecules is not clear from these characterizations most of the time (Fig. S2†), especially the ratio of copolymers to functional molecules.^42^ For example, signals of poly(AM-Rh*_x_ co* NIPAM*_y_*) in NMR spectra generate a broad peak, mainly belonging to P0, which is insufficient to identify information for AM-Rh (Fig. S3†). Considering that both poly(AM-Rh*_x_ co* NIPAM*_y_*) and AM-Rh have obvious colour characteristic absorption peaks, the characterization of poly(AM-Rh*_x_ co* NIPAM*_y_*) was attempted by UV-Vis spectroscopy. Results show that the maximum absorption peaks (*λ*_max_) for the four copolymers are nearly the same at 539 nm. They distinctly show *λ*_max_ of the mixture of P0 and AM-Rh centred at 542 nm, which is the same with the *λ*_max_ of monomer AM-Rh (Fig. 1a). This result indicates that AM-Rh is successfully covalently grafted onto the polymer chains. It also verifies that spectroscopy is an effective way to characterise polymers containing units with characteristic absorption.

The ratio (*x*/*y*) of respective units of poly(AM-Rh*_x_ co* NIPAM*_y_*) was therefore determined by combining UV-Vis spectroscopy and gel-permeation chromatography (GPC). The test method and processes are illustrated in detail in Fig. S4.† Firstly, the molar absorption coefficients (*ε*) of poly(AM-Rh*_x_ co* NIPAM*_y_*)s in open-ring form in methanol were estimated. Hereon, *ε* values for poly(AM-Rh*_x_ co* NIPAM*_y_*)s are approximately considered to be the same as the AM-Rh's *ε*, which was calculated to be 111 850 L mol^−1^ cm^−1^ by standard curve method according to Beer–Lambert law (Fig. S5†). Secondly, the number-averaged molecular weight (*M*_n_) of the poly(AM-Rh*_x_ co* NIPAM*_y_*)s was measured by gel permeation chromatography (GPC) (Fig. 1b and S6†). On the basis of *M*_n_ of poly(AM-Rh*_x_ co* NIPAM*_y_*), methanol solutions of poly(AM-Rh*_x_ co* NIPAM*_y_*) with a certain concentration (*C*_poly(AM-Rh_*_x co_*_NIPAM_*_y_*_)_) were obtained, and the absorbance (*A*_poly(AM-Rh_*_x co_*_NIPAM_*_y_*_)_) of the open-ring form of grafted AM-Rh was determined by UV-Vis spectroscopy. According to Beer–Lambert law and *ε*, the concentrations of grafted AM-Rh on poly(AM-Rh*_x_ co* NIPAM*_y_*) (*C*_AM-Rh__grafted on polymer_) were calculated, respectively. Then, the value of *x* was obtained from *C*_AM-Rh__grafted on polymer_/*C*_poly(AM-Rh_*_x co_*_NIPAM_*_y_*_)_, and the value of *y* was obtained from *M*_n__AM-Rh_*x* + *M*_n__NIPAM_*y* = *M*_n__poly(AM-Rh_*_x co_*_NIPAM_*_y_*_)_. Finally, the value of *x*/*y* for poly(AM-Rh*_x_ co* NIPAM*_y_*) was obtained as well (Table 1).

**Table 1:** Composition of the poly(AM-Rh*_x_ co* NIPAM*_y_*)

Polymer	*n*_AM-Rh_ : *n*_NIPAM_	*M*_n_	*Ð*	*x*	*y*	*x*/*y*
P0	0 : 100	332480	1.46	0	—	—
P1/50	1 : 50	148295	2.19	17	1213	1/71
P1/100	1 : 100	183190	1.26	11	1571	1/142
P1/200	1 : 200	395317	1.74	15	3430	1/229
P1/345	1 : 345	160250	2.17	4	1373	1/343

### Water solubility and hydrochromic property

**Fig. 2:**
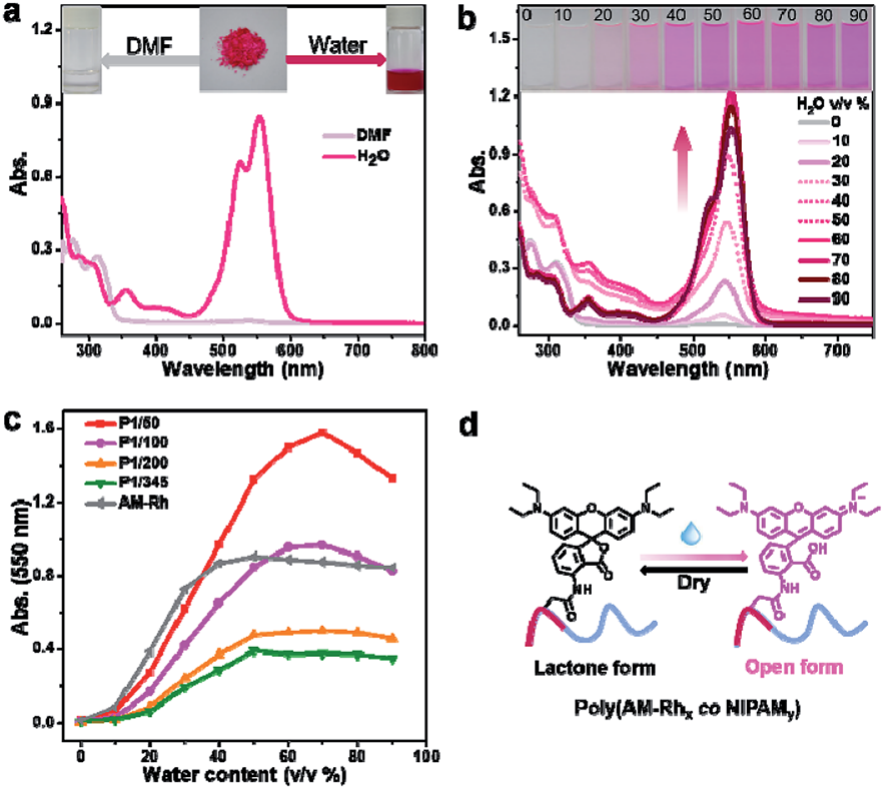
(a) UV-Vis spectra of P1/100 in water (0.2 mg mL^−1^) and DMF (0.2 mg mL^−1^), respectively. Inset: photos of P1/100 in water and DMF. (b) UV-Vis absorption spectra of P1/100 in variable mixtures of DMF and water with increasing percentage of water by volume from 0 to 90% (0.2 mg mL^−1^). Inset: photographs of the solution of P1/100 in variable mixtures of DMF and water with different amounts of water (from 0% to 90%). (c) Summarized water-content-dependent changes of P1/50, P1/100, P1/200, P1/345 (*C* = 1 × 10^−5^ mol L^−1^) AM-Rh for the absorption at *λ*_max_. (d) Illustration of reversible water-stimulated isomerization between the lactone form and open form of poly(AM-Rh*_x_ co* NIPAM*_y_*).

Solubility, especially water solubility, is of great significance in extending the application of materials in biological fields or industry. Therefore, water solubility of the prepared copolymers was firstly investigated. As expected, the four copolymers all have good water solubility. As an example, P1/100 can dissolve completely in water, at a solubility of more than 20 mg mL^−1^. In addition, it also has good solubility in most organic solvents, such as chloroform, methanol, ethanol, tertiary butanol, *N*,*N*-dimethyl formamide (DMF), tetrahydrofuran, dimethyl sulfoxide, acetonitrile, and acetone. As can be seen in Fig. 2a, P1/100 was dissolved in water, giving a magenta solution, and the main absorption band for the aqueous solution is between 450–600 nm, which is similar to the maximum absorption of AM-Rh in halochromism (Fig. S7†). The only difference is that the intensity of the shoulder peak around 524 nm for P1/100 is much higher (Fig. S8†). P1/100 dissolved in DMF solution is colourless, and the main absorption band is less than 350 nm. These results indicate that P1/100 exists in lactone form in DMF, while it exists in open-ring form in water. That is, in addition to water solubility, P1/100 maintains its hydrochromic property.

In order to investigate the hydrochromic property in detail, the colour change and corresponding UV-Vis spectra of P1/100 in the DMF solution with increasing water content were examined. As shown in Fig. 2b, the colourless DMF solution of P1/100 gradually changed to magenta with the increase of water content, and the absorption peak intensity at around 550 nm, assigned to the main absorption of its open-ring form, also gradually increased with the increase of water content until it reached 70%. With further increase in water content (80% and 90%), the absorption intensity at around 550 nm decreased, accompanied by the increase of absorption intensity for the shoulder peak at around 524 nm. Combined with the absorption spectra of P1/100 in pure water, it can be concluded that the shoulder peak is caused by hydration of the open-ring form of AM-Rh.^23^ It is also worth mentioning that no isosbestic point was observed for these UV-Vis spectra, which is different from our previously reported hydrochromic dyes.^8,23^ This should be caused by irregular absorption changes of the polymer with varying compositions of the mixed solvents, especially in the binary solvents with water content of 30%, 40% and 50%, in which the copolymer has poor solubility and showed increasing absorption around 250–500 nm. To prove this point, absorption spectra of P0 under the same condition was detected. The obvious poor solubility was also clearly observed in the binary solvents with water content of 30%, 40% and 50% both in UV-Vis spectra and pictures (Fig. S9 and S10†). Moreover, the spectra for P1/100 in pure DMF with gradual addition of acid and for AM-Rh in DMF-H_2_O binary solvents with varying water content all have isosbestic points (Fig. S11 and S12†), which further demonstrates our point.

In order to further investigate whether the ratio of dye molecules grafted to the polymer has an effect on their hydrochromism, the three other copolymers of poly(AM-Rh*_x_ co* NIPAM*_y_*), that is, P1/50, P1/200 and P1/345, were also tested under the same conditions and compared to P1/100 and AM-Rh. From the curves of their absorbance at 550 nm with changing water content, we can observe that the four copolymers grafted with AM-Rh all retained their hydrochromic property, while their hydrochromic sensitivity (reflected by slope of the curve changes) decreased, compared to the monomer AM-Rh. In addition, among the four copolymers, as the dye graft rate decreases, their hydrochromic sensitivity decreases, that is, P1/50 > P1/100 > P1/200 > P1/345 (Fig. 2c). Furthermore, the maximum absorption intensity for their hydrochromism can also be adjusted by changing the grafting ratio of AM-Rh to NIPAM. That is, the maximum absorption intensity increases gradually with grafting ratio of AM-Rh to NIPAM. Structural change of the dye molecule-grafted poly(AM-Rh*_x_ co* NIPAM*_y_*) corresponding to hydrochromism, is shown in Fig. 2d.

### The fluorescence property of Poly(AM-Rh*_x_ co* NIPAM*_y_*)

**Fig. 3:**
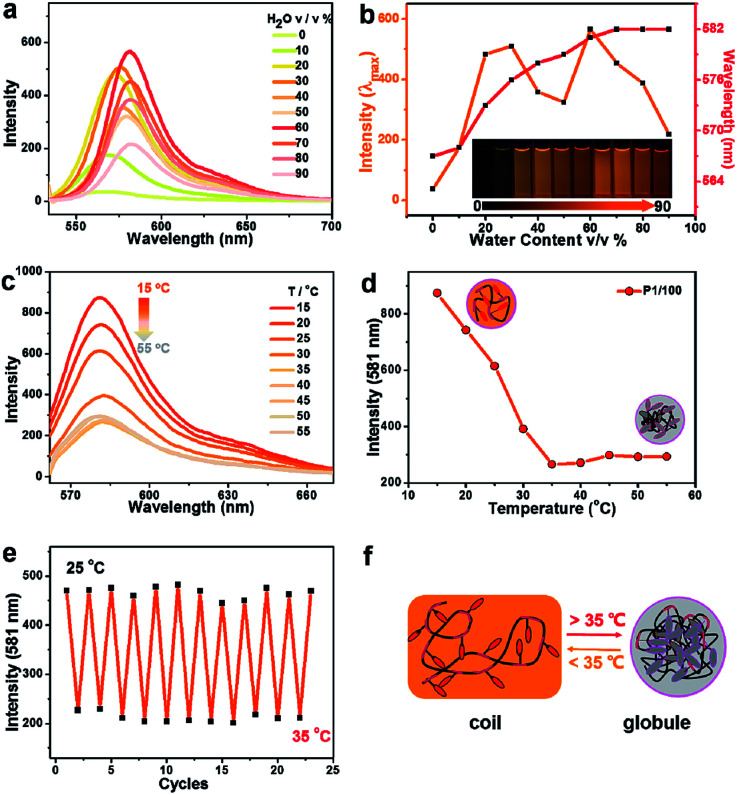
(a) Fluorescence spectra of P1/100 in variable mixtures of DMF and water, with increasing percentage of water by volume from 0 to 90% (*C* = 1 × 10^−5^ mol L^−1^, *λ*_ex_ = 530 nm; slit width: 3, 1.5, 25 °C). (b) Plot of fluorescence intensity at *λ*_max_ with water content and corresponding maximum emission wavelength (*λ*_ex_ = 530 nm; slit width: 3, 1.5; 25 °C). Inset: Photographs of the solution of P1/100 (0.2 mg mL^−1^) in variable mixtures of DMF and water with different amounts of water, from 0% to 90%. (c) Fluorescence spectra of P1/100 (0.2 mg mL^−1^) in water with varying temperature from 15 °C to 55 °C. (d) Temperature-dependent change in fluorescence (*λ*_ex_ = 550 nm; slit width: 1.5, 3) of P1/100 (0.2 mg mL^−1^) in water, with temperature varied from 15 °C to 55 °C. (e) A plot of the fluorescence at 581 nm *versus* the number of cycles as P1/100 is cycled through heating and cooling between 25 °C to 35 °C (*λ*_ex_ = 550 nm; slit width: 1.5, 3). (f) Schematic for fluorescence intensity variation of poly(AM-Rh*_x_ co* NIPAM*_y_*) in water between 25 °C to 35 °C.

Fluorescence properties of poly(AM-Rh*_x_ co* NIPAM*_y_*)s were also investigated. Hereon, P1/100 is also described as an example. Firstly, the fluorescence of P1/100 in DMF-H_2_O binary solutions with varying water content was studied. As shown in Fig. 3a, both the intensity and maximum emission peak of the fluorescence spectra for P1/100 have changed with increasing percentage of water. The generation of fluorescence mainly comes from the ring opening of the lactone ring of AM-Rh to form a conjugation-extended open-ring structure (Fig. S13†). The fluorescence signals for both poly(AM-Rh*_x_ co* NIPAM*_y_*)s and AM-Rh are stronger than NH_2_-Rh under the same conditions (Fig. S14†). The emission peak position gradually red shifted with the increase of water content (Fig. 3b, red line). This is due to the solvation effect on the zwitterionic open-ring form of AM-Rh, whose ground state dipole moment is less than its excited state dipole moment (*μ*_g_ < *μ*_e_).^23,43^

The cause of fluorescence in poly(AM-Rh_*x*_*co* NIPAM_*y*_)s was further proved by the fact that similar changes of peak position were observed in AM-Rh under the same conditions (Fig. S15†). Meanwhile, the change trend of P1/100 with increased water content presents an M-shaped (Fig. 3b, orange line). From 0–30%, the fluorescence intensity increases with increasing water content, which is mainly due to the gradual increase of ratio for the open-ring form of AM-Rh; at 30–50%, the intensity decreased gradually, which may be mainly due to the reduction of solubility for P1/100 in the binary solutions as we discussed above; from 60%–90%, the intensity decreased again, despite increasing at 60% due to decrease of absorbance for the open-ring form of AM-Rh, as shown in Fig. 2c (70–90%).

Given that poly(*N*-isopropyl acrylamide) is a water-soluble thermo-responsive polymer that undergoes temperature-regulated, reversible coil-to-globule phase transition, the capability of poly(AM-Rh*_x_ co* NIPAM*_y_*) to achieve tunable fluorescence with temperature variation in water was verified next. Fluorescence spectra change of P1/100 in water was measured by varying the temperature range from 15 °C to 55 °C (Fig. 3c). P1/100 is soluble in water (coil), with distinct fluorescence at low temperature (15 °C), but a rise in temperature leads to a gradual fluorescence quenching (Fig. 3d). When temperature is up to 35 °C, the fluorescence intensity reached the lowest, remaining the same as the temperature continued to increase (Fig. 3d). This is presumably due to the formation of copolymer particles^44,45^ associated with the aggregation of copolymer chain (globule) at temperatures exceeding 35 °C. In addition, the decrease in fluorescence intensity due to intermolecular interaction enhanced by increasing temperature is one of the reasons (Fig. S14†). Furthermore, this tunable fluorescence intensity change for P1/100, adjusted by increasing and reducing temperature, is reversible and repeatable. As shown in Fig. 3e, nearly no intensity decrease was observed after ten consecutive heating–cooling cycles. The corresponding schematic diagram for fluorescence intensity and state changes of the copolymer chain between the coil form at low temperature (<35 °C) and globule form at high temperature (>35 °C) is illustrated in Fig. 3f.

**Fig. 4:**
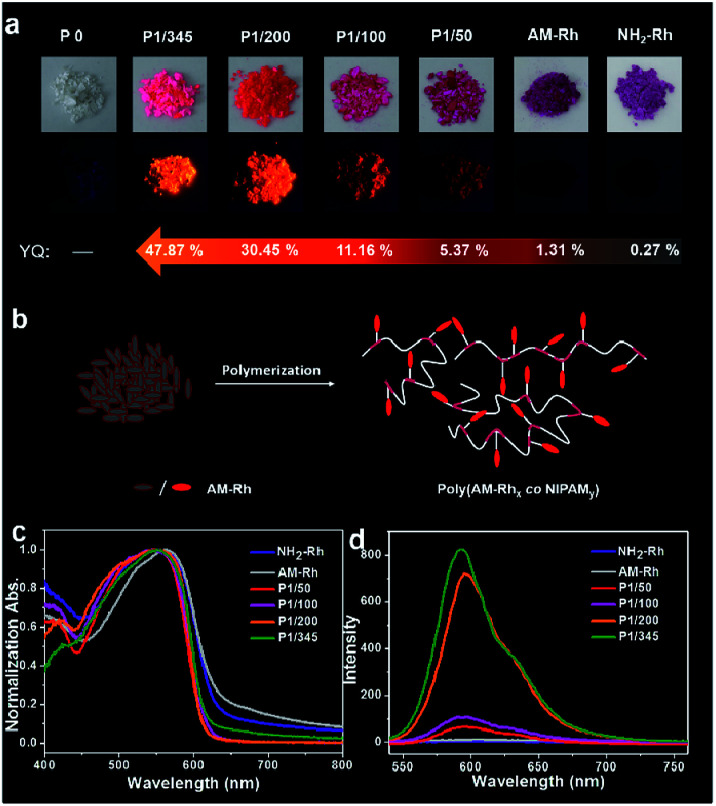
(a) Photos of the powders of P0, P1/345, P1/200, P1/100, P1/50, AM-Rh, and NH_2_-Rh under room light and UV light of 365 nm, respectively, and their corresponding quantum yields (QYs). (b) Schematic for the fluorescence emission of AM-Rh- and AM-Rh-grafted poly(AM-Rh*_x_ co* NIPAM*_y_*) in powder form. (c) Normalized UV-Vis spectra and (d) fluorescence spectra of P1/345, P1/200, P1/100, P1/50, AM-Rh, and NH_2_-Rh in powder form (*λ*_ex_ = 530 nm; slit width: 3, 1.5).

Fluorescence properties of the four poly(AM-Rh*_x_ co* NIPAM*_y_*)s in solid were also studied. These four polymers have fluorescence emission under solid powder; nevertheless, there is nearly no emission observed for powders of AM-Rh or NH_2_-Rh (Fig. 4a). The fluorescence emission fluorescence comes from the open-ring form of AM-Rh in the poly(AM-Rh*_x_ co* NIPAM*_y_*), which is verified by the powder colour of magenta and the main absorption peak around 500–600 nm *via* UV-Vis spectroscopy in Fig. 4c. Their fluorescence intensities were qualitatively measured (spectra shown in Fig. 4d) and quantitatively tested by quantum yields (QYs). QYs for the powders of P1/345, P1/200, P1/100, P1/50, AM-Rh, and NH_2_-Rh are 47.87%, 30.45%, 11.16%, 5.37%, 1.31%, and 0.27%, respectively, as shown in Fig. 4a. The low QYs of AM-Rh and NH_2_-Rh are due to aggregation-caused quenching (ACQ).^46–48^

The high QYs of poly(AM-Rh*_x_ co* NIPAM*_y_*) are due to the dispersal effect of the polymer chains preventing molecular aggregation. The fluorescence QYs for the four poly(AM-Rh*_x_ co* NIPAM*_y_*)s (that is, P1/345, P1/200, P1/100, P1/50) decrease as the AM-Rh/NIPAM ratio decreases, which further indicates that ACQ could be effectively prevented by adjusting the proportion of dyes grafted into the polymer. This also means that grafting of ACQ molecules into polymers at appropriate proportions is an effective way to achieve their solid-state luminescence (Fig. 4b).

## Conclusion

In summary, the properties of a hydrochromic material in terms of water solubility and adjustable fluorescence in both solution and solid state are successfully improved by grafting it to a thermosensitive water-soluble polymer. Four poly(AM-Rh*_x_ co* NIPAM*_y_*)s with different dye grafting ratios were obtained by changing the raw ratio of copolymerization. To address the insufficiency of commonly used characterization methods for dye-grafted copolymers, in this work, we put forward a more effective method *via* UV-Vis absorption spectroscopy and provide detailed methods and steps for calculating dye-grafting ratio (*x*/*y*) by spectroscopy combined with GPC technologies. The four copolymers have good solubility in many common organic solvents and even in pure water, meanwhile preserving the hydrochromism of the dye monomer. It was found that the difference in grafting ratio not only influences the maximum absorbance of the colour, but also affects the sensitivity of their hydrochromism. Based on aggregation and dispersion of the four poly(AM-Rh*_x_ co* NIPAM*_y_*)s as adjusted by solvent media and temperature, the reversible regulation of their fluorescence properties was successfully realized. In addition, their luminescence in solid state was enabled by this covalent grafting strategy, which effectively overcomes the aggregation-induced fluorescence quenching of dyes. These studies are of great significance for expanding the application of hydrochromic dye molecules in biological fields and promoting green industrialization.

## Experimental section

### Materials

3-Nitrophthalic anhydride (98%), *N*,*N*-diethyl-3-aminophenol (98%), Pd/C (10%), *o*-phthalic anhydride (98%), and acryloyl chloride (97%) were purchased from Energy Chemical (Shanghai, China). *Tert*-butyl alcohol was purchased from East China Reagent (Tianjin, China). Azodiisobutyronitrile (AIBN) was purchased from Aladdin (Shanghai, China). Methanol (99.9%) and *N*,*N*-dimethyl formamide (DMF) (99.9%) were purchased from Yuwang Reagent. Dialysis membrane was purchased from MYM Biological Technology Company Limited. Unless otherwise noted, all the other materials were purchased from Sinopharm Chemical Reagent Beijing Co. without further purification. Deionized water was purified by Milli-Q system.

### Instruments

Absorption spectra were measured using a Shimadzu UV-2550 PC double-beam spectrophotometer. Absorption spectra of solids were measured *via* the reflective mode of the integrating sphere on an Analytik Jena Specord®210 plus UV/Vis spectrophotometer, using BaSO_4_ as background and path length of 1 cm. The fluorescence quantum yields (QYs) were measured on a FLS 920 lifetime and steady-state spectrometer. Steady-state fluorescence spectra were measured using a Shimadzu RF-5301 PC spectrophotometer. ^1^H NMR (500 MHz) and ^13^C NMR (126 MHz) spectra were recorded on a Bruker AVANCE 500 at room temperature. LC-HRMS analysis was performed on an Agilent 1290-Micro TOF-Q II mass spectrometer. *M*_n_ and *M*_w_/*M*_n_ (*Ð*) of poly(AM-Rh*_x_ co* NIPAM*_y_*)s were measured using PL-GPC 220 high-temperature gel permeation chromatography (GPC) with Agilent PLgel MIXED-B (300 × 7.5 mm, 10 μm), taking DMF as the mobile phase with flow rate of 1.0 mL min^−1^ at 80 °C, and using RI as the detector and polystyrene (PS) as calibration standard.

### Synthesis/preparation of NH_2_-Rh, AM-Rh and Poly(AM-Rh*_x_ co* NIPAM*_y_*)s

NH_2_-Rh was prepared according to reported literature.^49^

#### Synthesis of AM-Rh

NH_2_-Rh (1 mmol, 457 mg) was dissolved in CH_2_Cl_2_ (10 mL), and Et_3_N (2 mmol, 270 μL) and acryloyl chloride (1.2 mmol, 98 μL) were added dropwise to the solution at 273 K under dry N_2_ and stirred at 343 K for 10 h. The resulting solution was washed with 1 M HCl and saturated NaHCO_3_ solutions, dried over Na_2_SO_4_, and concentrated by evaporation. The product was purified by column chromatography (silica, petroleum ether : ethyl acetate : methanol is 5 : 5 : 1), yielding a bright red solid powder (270 mg, 52%). ^1^H NMR (500 MHz, DMSO) *δ* 10.23 (s, 1H), 8.44 (d, *J* = 8.2 Hz, 1H), 7.73 (t, *J* = 7.9 Hz, 1H), 6.92 (d, *J* = 7.5 Hz, 1H), 6.63–6.55 (m, 3H), 6.48–6.44 (m, 4H), 6.37 (d, *J* = 17.0 Hz, 1H), 5.92 (d, *J* = 10.4 Hz, 1H), 3.37 (q, *J* = 6.8 Hz, 8H), 1.11 (t, *J* = 7.0 Hz, 12H). LC-HRMS: *m*/*z* calculated for [M + H]^+^ 512.2544, found 512.2536.

#### Synthesis of P1/50

AM-Rh (0.1 mmol, 51 mg) and *N*-isopropylacrylamide (NIPAM, 5 mmol, 565 mg) were dissolved in tertiary butanol (*t*-BuOH) (5 mL) under the protection of N_2_. The solution was cooled by liquid nitrogen, azodiisobutyronitrile (AIBN) (6 mg, 1%) was added, and the mixture was stirred at 343 K for 12 h under N_2_. The polymer was concentrated by evaporation and purified by dialysing with methanol in a dialysis bag with a cutoff of 3500 for 60 h. The polymer formed was precipitated with methanol (3 mL) and diethyl ether (200 mL). After drying *in vacuo*, P1/50 was obtained as a dark pink solid (540 mg, 90%). ^1^H NMR (500 MHz, CDCl_3_) *δ* (ppm) 3.99 (s, 1H), 3.15 (s, 0.5H), 2.47–1.47 (m, 3H), 1.12 (s, 6H).

#### Synthesis of P1/100

AM-Rh (0.1 mmol, 51 mg) and NIPAM (10 mmol, 1.2 g) were dissolved in *t*-BuOH (8 mL) under the protection of N_2_. The solution was cooled by liquid nitrogen, then AIBN (12 mg, 1%) was added and the mixture stirred at 343 K for 12 h under N_2_. The purification process is the same as that for P1/50. P1/100 was obtained as a pink solid (1.06 g, 89%).

#### Synthesis of P1/200

AM-Rh (0.05 mmol, 25 mg) and NIPAM (10 mmol, 1.2 g) were dissolved in *t*-BuOH (6 mL) under the protection of N_2_. The solution was cooled by liquid nitrogen, then AIBN (12 mg, 1%) was added and the mixture stirred at 343 K for 12 h under N_2_. The purification process is the same as that for P1/50. P1/200 was obtained as a pink solid (1.10 g, 92%).

#### Synthesis of P1/345

AM-Rh (0.04 mmol, 20 mg) and NIPAM (13.2 mmol, 1.5 g) were dissolved in *t*-BuOH (9 mL) under the protection of N_2_. The solution was cooled by liquid nitrogen, then AIBN (16 mg, 1%) was added and the mixture stirred at 343 K for 12 h under N_2_. The purification process is the same as P1/50. P1/345 was obtained as a light pink solid (1.31 g, 87%).

#### Synthesis of P0

NIPAM (8.8 mmol, 1.0 g) was dissolved in *t*-BuOH (6 mL) under the protection of N_2_. The solution was cooled by liquid nitrogen, then AIBN (10 mg, 1%) was added and stirred at 343 K for 12 h under N_2_. The purification process is the same as P1/50. P0 was obtained as a white solid (0.88 g, 88%).

## Conflicts of interest

There are no conflicts to declare.

## Supplementary Material

RA-008-C8RA01306C-s001
