# Supplementary material for: Water-soluble and adjustable fluorescence copolymers containing a hydrochromic dye: synthesis, characterization and properties
Source: RSC Adv. 2018 Apr 11;8(25):13664–70. doi: 10.1039/c8ra01306c (PMC9079821; doi:10.1039/c8ra01306c)
Supplement: RA-008-C8RA01306C-s001 [file RA-008-C8RA01306C-s001.pdf]

Electronic Supplementary Material (ESI) for RSC Advances.
This journal is © The Royal Society of Chemistry 2018

Supporting Information

Water-soluble and fluorescence adjustable copolymers containing a hydrochromic dye: synthesis, characterization and properties

Le Ju[a,b], Tianyou Qin[a], Ting Zhang[c], Peng Wang[a], Lan Sheng*[a] and Sean Xiao-An Zhang[b,c]

[a]College of Chemistry, Jilin University, Changchun, 130012, China.

E-mail: shenglan17@jlu.edu.cn; Fax: +86-431-85153812

[b]Department of Chemistry and Pharmacy, Zhuhai College of Jilin University, Zhuhai, 519041, China.

[c]State Key Lab of Supramolecular Structure and Materials, College of Chemistry, Jilin University, Changchun, 130012, China.

Table of Contents

1. NMR and IR spectra of AM-Rh and **poly(AM-Rh$_x$ *co* NIPAM$_y$)s (Figugre S1 – S4)**

2. Test and calculation of molar absorption coefficient of AM-Rh (**Figure S5)**

3. GPC raw date (**Figure S6**)

4. The contrast between halochromism of AM-Rh and hydrochromism (**Figure S7, S8)**

5. The solubility of **P0** in binary solvents of DMF-H_2O (**Figure S9, S10)**

6. Halochromism of **P1/100** and hydrochromic of AM-Rh in binary solvents of DMF-H_2O (**Figure S11, S12)**

7. Halochromism and florescence of AM-Rh in DMF (**Figure S13)**

8. Hydrochromism and florescence of NH_2-Rh in binary solvents of DMF-H_2O (**Figure S14)**

9. Florescence of AM-Rh in binary solvents of DMF-H_2O (**Figure S15)**

10. Florescence of AM-Rh vary with temperature (**Figure S16)**

1. NMR and IR spectra of AM-Rh and poly(AM-Rh$_x$ *co* NIPAM$_y$)s

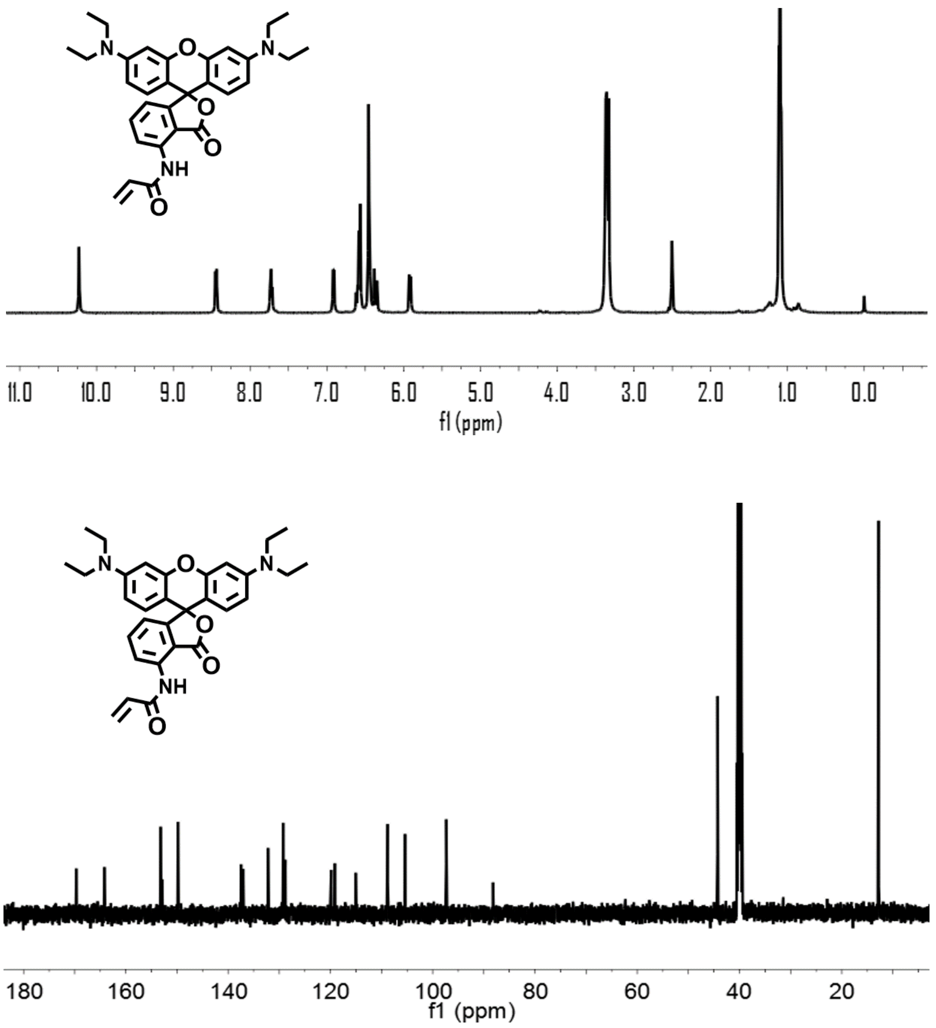

Figure S1. ^{1}H NMR and ^{13}C NMR of AM-Rh.

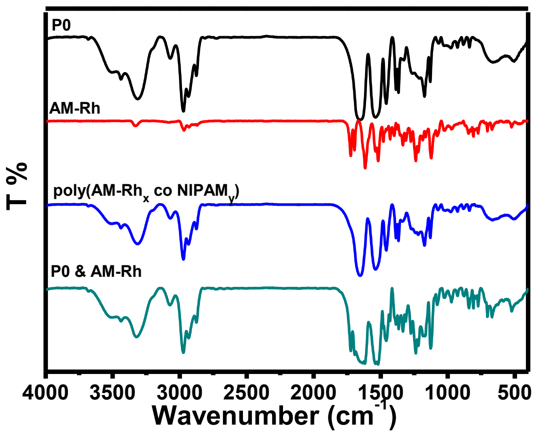

Figure S2. Infrared spectrum of **P0**, AM-Rh, **P1/50** and mixture of **P0** & AM-Rh.

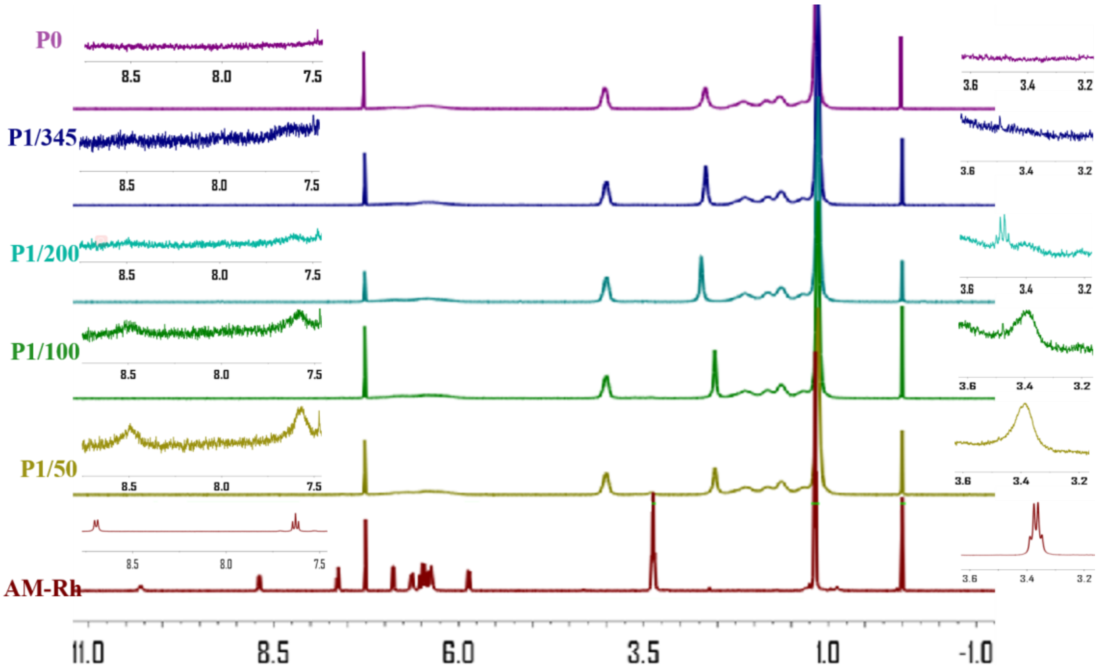

Figure S3. ^{1}H NMR spectrums of **P1/50, P1/100, P1/200, P1/345, P0**.

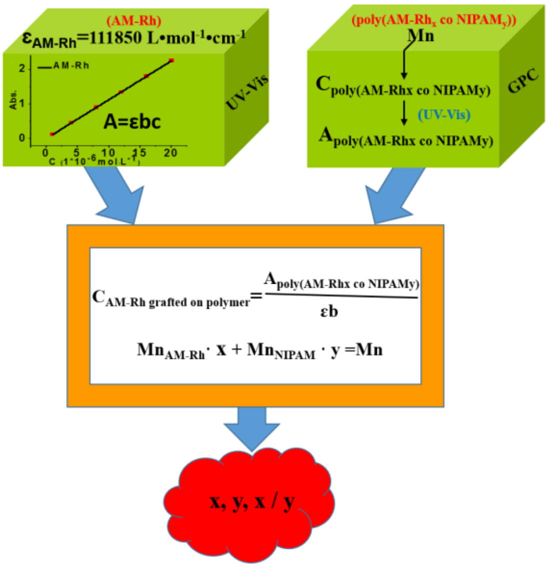

Figure S4 Schematic diagram for calculating values of x, y, and x / y.

2. Test and calculation of molar absorption coefficient of AM-Rh

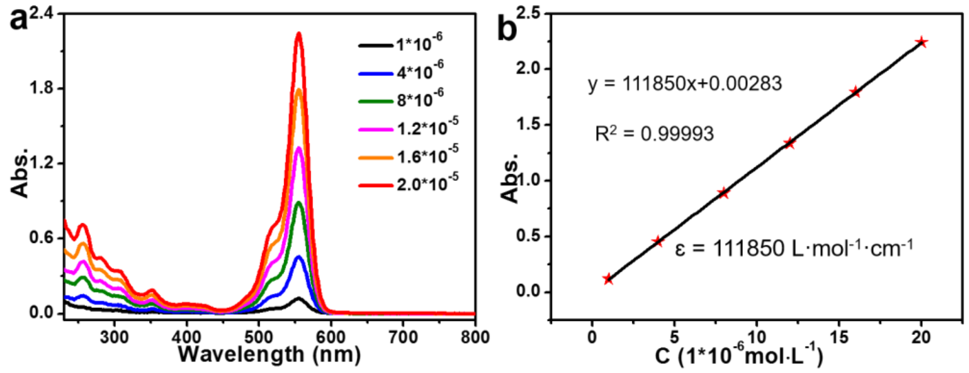

Figure S5. a) The UV-Vis spectra of AM-Rh in methol with concentration ranging from 1 $\times 10^{-6}$ mol L^{-1} to 2 $\times 10^{-5}$ mol L^{-1}. b) Absorbance values plotted against concentration in MeOH, molar absorption coeffcient (ε) of AM-Rh was calculated as 111850 L mol^{-1} cm^{-1}.

3. GPC raw date

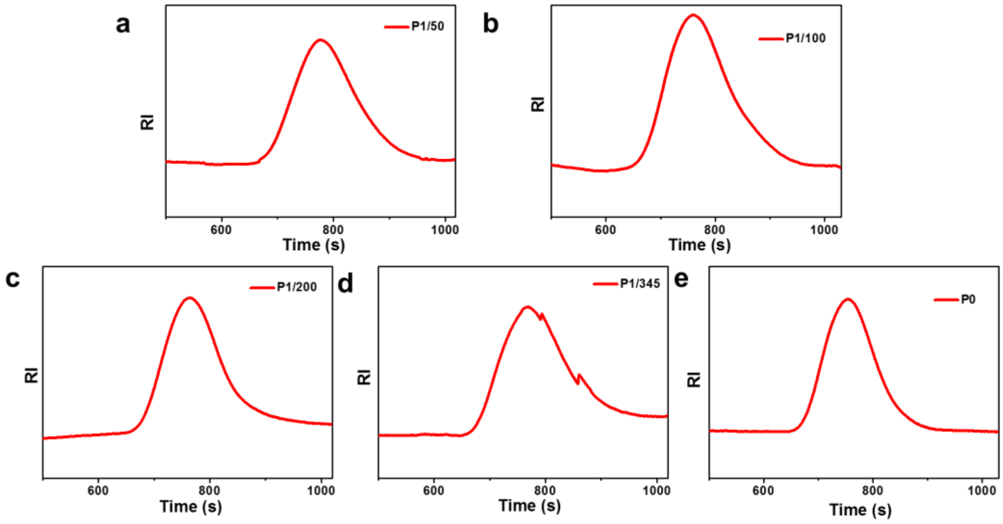

Figure S6. The raw GPC data of polymer.

4. The contrast between halochromism of AM-Rh and hydrochromism

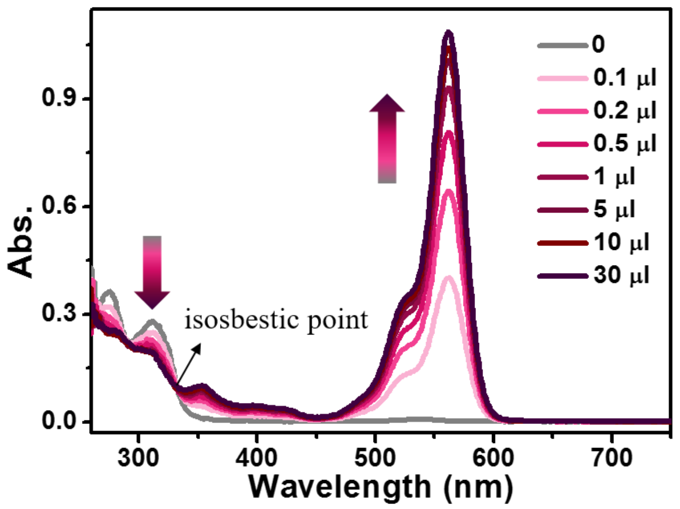

Figure S7. The UV-Vis spectra of AM-Rh (C = 1 $\times 10^{-5}$ mol L^{-1}) in DMF with gradually adding CF_3COOH.

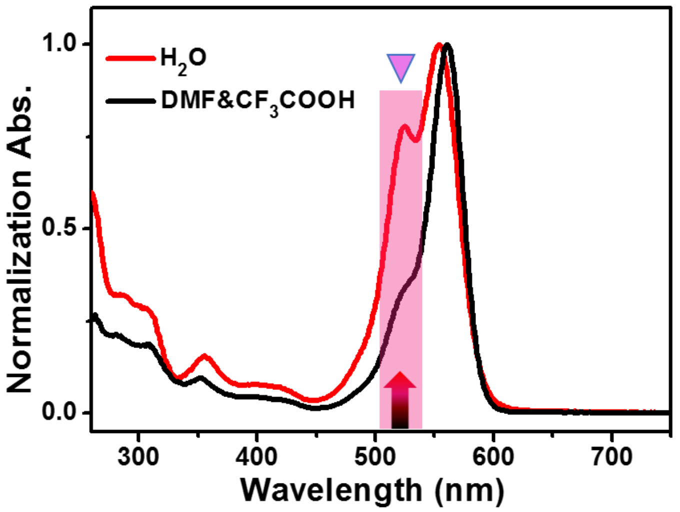

Figure S8. Normolized UV-Vis spectra of the solution of **P1/100** (0.2 mg / mL) in water and in DMF with addition of CF_3COOH.

5. The solubility of P0 in DMF-H_2O mixed systems

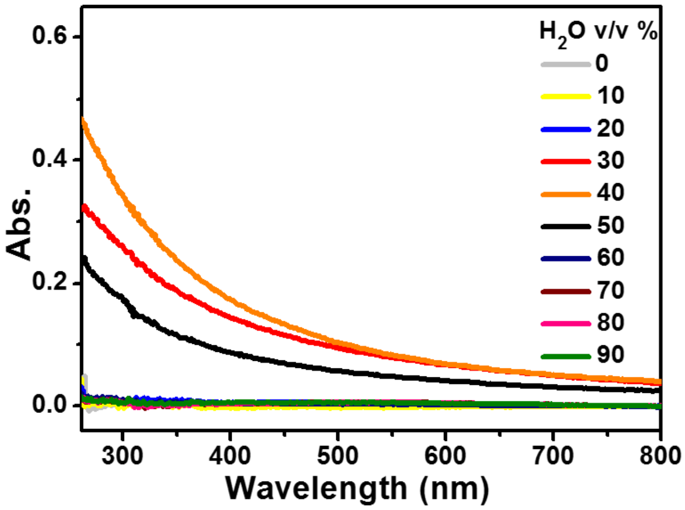

Figure S9. The UV-Vis spectra of the solution of **P0** (0.2 mg / mL) in variable mixtures of DMF and water with increasing percentage of water by volume from 0 to 90% at 25 °C.

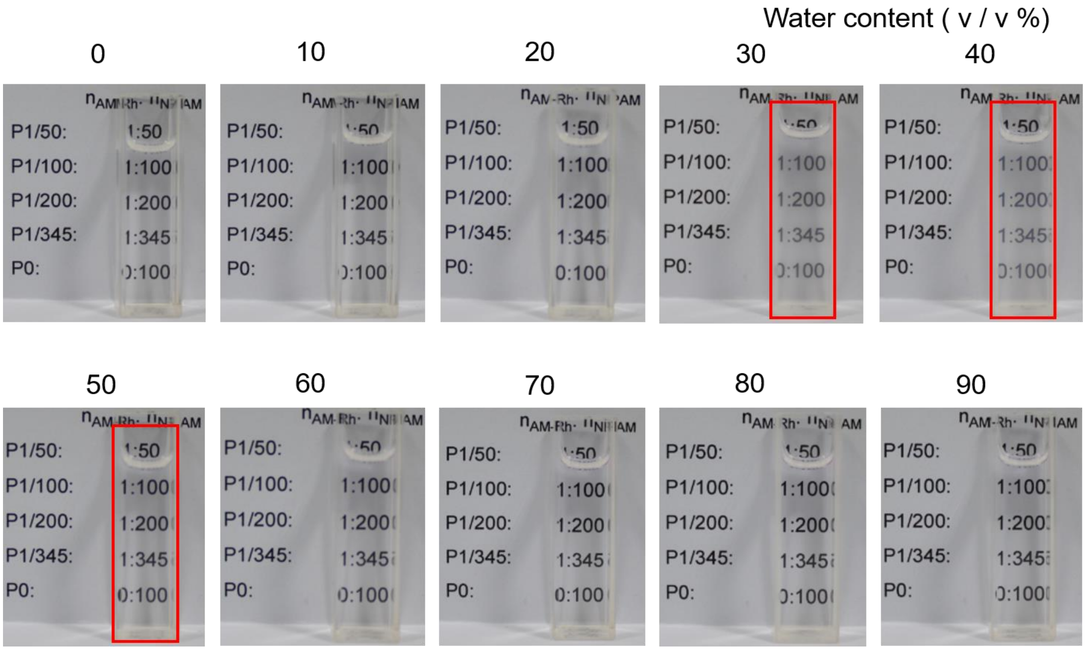

Figure S10. Photographs of **P0** (0.2 mg / mL) in variable mixtures of DMF and water with increasing percentage of water by volume from 0 to 90% at 25 °C.

6. Halochromism of P1/100 and hydrochromic of AM-Rh in mixtures of DMF-H_2O

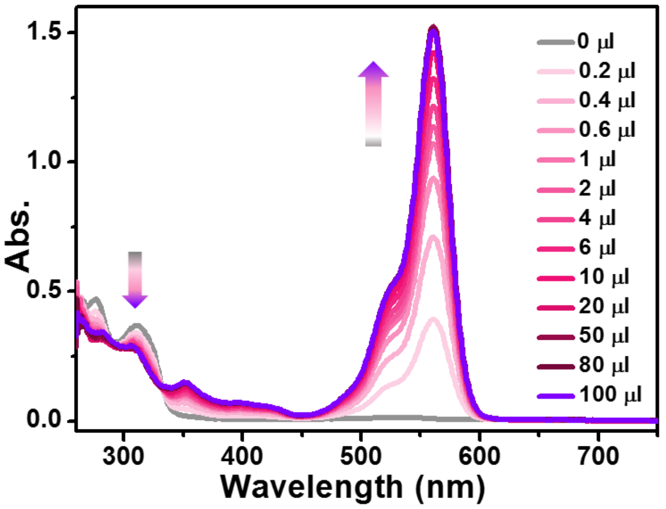

Figure S11. The UV-Vis spectra of **P1/100** (0.2 mg / ml) in DMF with adding CF_3COOH.

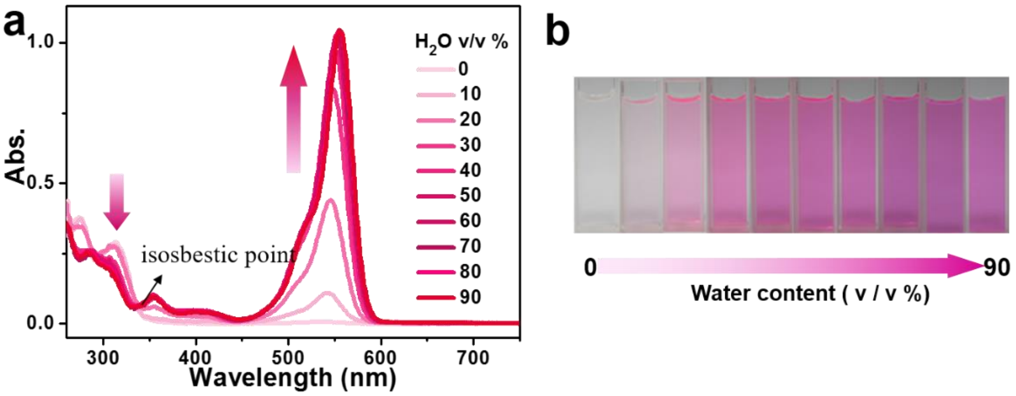

Figure S12. a) UV-Vis spectra of the solution of AM-Rh (C = 1 × 10^{-5} mol L^{-1}) in variable mixtures of DMF and water with increasing percentage of water by volume from 0 to 90%. b) The corresponding photographs of AM-Rh in different water contents.

7. Halochromism and florescence of AM-Rh in DMF with adding CF_3COOH

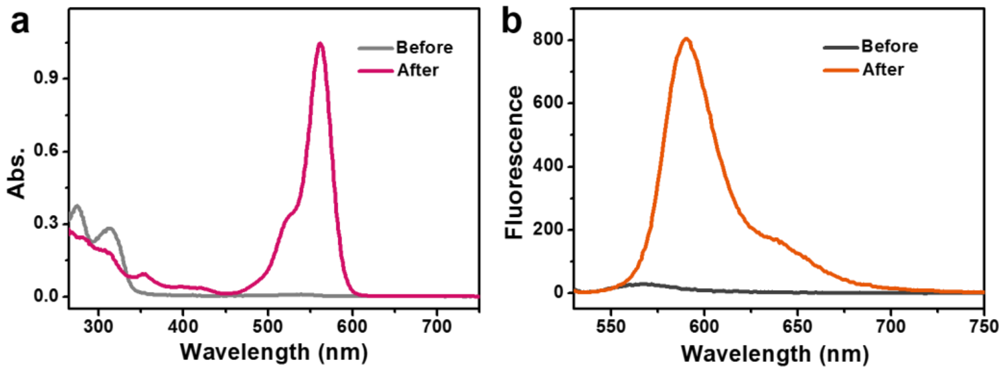

Figure S13. a) The UV-Vis spectra of AM-Rh (C = 1 × 10^{-5} mol L^{-1}) in DMF before (gray) and after (magenta) adding CF_3COOH. b) Fluorescence of AM-Rh (C = 1 × 10^{-5} mol L^{-1}) in DMF before (gray) and after (orange) adding CF_3COOH (λ_{ex} = 530 nm; slit width: 3, 1.5).

8. Hydrochromic and florescence of NH_2-Rh in mixtures of DMF-H_2O

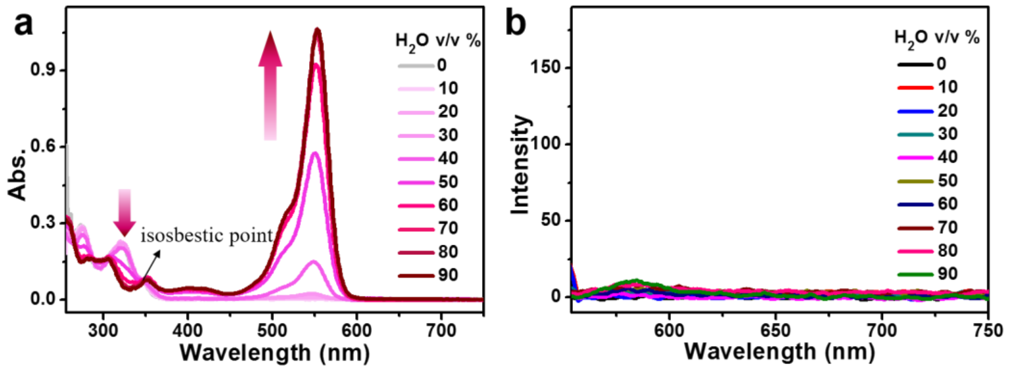

Figure S14. a) UV-vis spectra of the solution of NH_2-Rh (C = 1 × 10^{-5} mol L^{-1}) in variable mixtures of DMF and water with increasing percentage of water by volume from 0 to 90%. b) The fluorescence spectra of the solution of NH_2-Rh in variable mixtures of DMF and water with increasing percentage of water by volume from 0 to 90% at 25 °C (C = 1 × 10^{-5} mol L^{-1}, λ_{ex} = 530 nm; slit width: 3, 1.5).

9. Florescence of AM-Rh in mixtures of DMF-H_2O

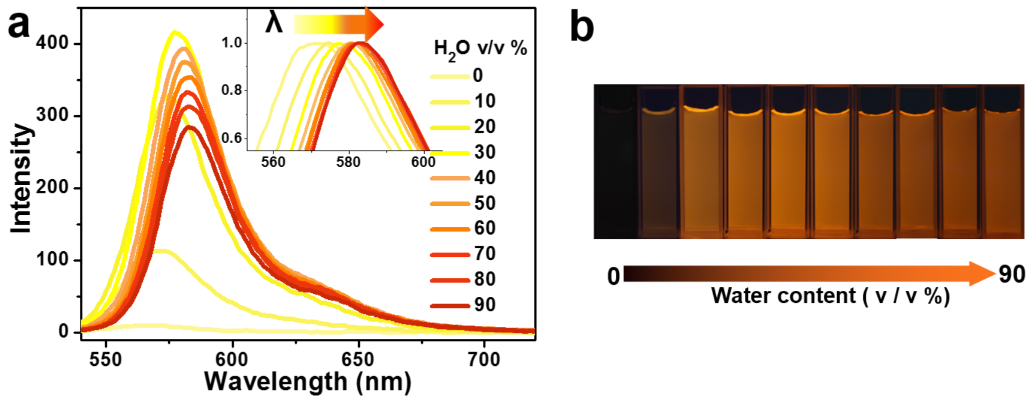

Figure S15. a) The fluorescence spectra of the solution of AM-Rh in variable mixtures of DMF and water with increasing percentage of water by volume from 0 to 90% at 25 °C (C = 1 × 10^{-5} mol L^{-1}, λ_{ex} = 530 nm; slit width: 3, 1.5). b) The corresponding fluorescence photographs of AM-Rh in different water contents.

10. Florescence of AM-Rh vary with temperature

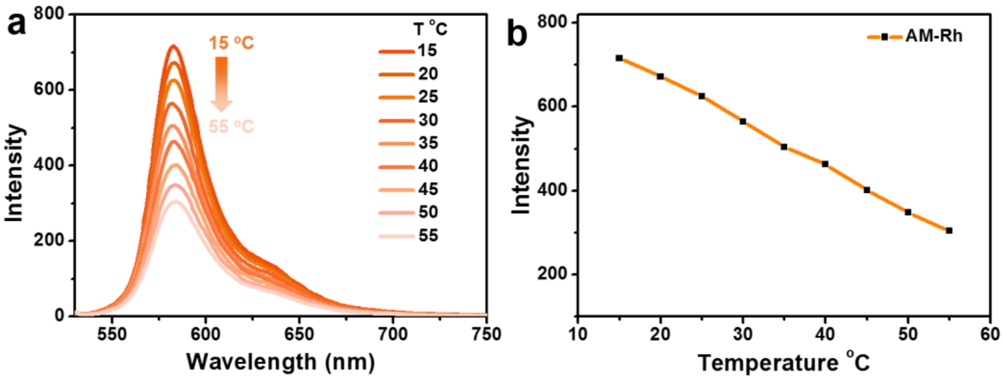

Figure S16. a) The fluorescence spectra of the solution of AM-Rh (C = 1 × 10^{-5} mol L^{-1}) in H_2O with varying temperature from 15 to 55 °C (λ_{ex} = 530 nm; slit width: 3, 1.5). b) Fluorescence changes of AM-Rh (C = 1 × 10^{-5} mol L^{-1}) in H_2O with varying temperature from 15 to 55 °C.
